# Correlation of vitamin D receptor gene (ApaI) polymorphism with periodontitis: A meta‐analysis of Chinese population

**DOI:** 10.1002/fsn3.1215

**Published:** 2019-09-27

**Authors:** Hai‐xia Guo, Jian Pan, Heng‐biao Pan, Si‐jia Cui, Chun‐ying Fang

**Affiliations:** ^1^ Department of Stomatology Zhenhai Longsai Hospital Ningbo China; ^2^ Department of Stomatology, Ningbo No.7 Hospital Ningbo China

**Keywords:** meta‐analysis, periodontitis, polymorphism, VDR ApaI

## Abstract

Many studies have tried to elucidate the connection between vitamin D receptor (VDR) gene (ApaI) polymorphism and periodontitis; however, so far there is no consensus. To further assess the impact of ApaI polymorphism on periodontitis risk, we have conducted a meta‐analysis of Chinese population. Relevant literatures were searched according to PubMed and Chinese database in January 2019. The strength of correlation was evaluated by combining odds ratio (ORs) and 95% confidence interval (CIs). Six case–control studies were identified with inclusion criteria, including 734 cases of periodontitis and 687 controls. Based on the overall analysis, the VDR ApaI polymorphism was not due to the risk of periodontitis in all models. Subgroup analysis showed that the risk of periodontitis in North China was significantly reduced. To sum up, the study shows that VDR‐ApaI polymorphism may be connected with a lower risk of periodontitis in northern China. It is suggested that inferential studies should be conducted in other ethnic groups.

## 1 INTRODUCTION

Periodontitis major is composed of chronic periodontitis (CP) and invasive periodontitis (AP) (Armitage, 1999). Periodontitis major is distributed widely in many countries, which is the main cause of tooth loss in the elderly. Therefore, in order to enhance the quality of life of the elderly, it is of great significance to elucidate the pathogenesis of the disease. A few years ago, vitamin D receptor (VDR) gene polymorphism was considered to be an important factor in regulating bone metabolism, which attracted wide attention as a risk factor for osteoporosis (Eckstein et al., 2002; Kung, Yeung, & Lau, 1998; Tantawy, Amer, Raafat, & Hamdy, 2016; Tokita et al., 1996). It is reported that VDR gene contributes to the occurrence of tuberculosis (Wilkinson et al., 2000). This finding may be interpreted as indicating that VDR gene polymorphism is closely related to the immune function of monocytes, because vitamin D is thought to contribute to the phagocytosis of monocytes. In recent years, many studies have tried to elucidate the connection between VDR gene ApaI polymorphism and periodontitis, but so far there is no consensus. Differences in outcomes may be connected to the studied patients’ racial and clinical heterogeneity or to the relatively small number. A meta‐analysis is a method to overcome the problem of small sample size and inadequate statistical ability. To better address the relationship between VDR ApaI polymorphism and periodontitis risk, we conducted a meta‐analysis of Chinese population to reduce the impact of different genetic backgrounds.

## 2 MATERIALS AND METHODS

### 2.1 Search strategy and selection criteria

Applying the PubMed and Chinese databases to search for articles about the correlation between VDR ApaI gene polymorphism and periodontitis through January 2019. The keyword combinations used in the search are as follows: (vitamin D receptor or VDR) and (periodontitis or periodontal disease) and (China or mainland China or Taiwan). Searches are conducted without language restrictions, with emphasis on human research. References cited in the retrieved articles are also included.

The criteria for inclusion are as follows: (a) case–control or cohort studies describing the correlation between VDR ApaI polymorphism and periodontitis; (b) genotype data of the VDR ApaI polymorphism in periodontitis patients and controls; and (c) participants are Chinese. The criteria for exclusion are as follows: (a) redundant literature; (b) absence of controls; (c) incomplete data; and (d) meta‐analyses, letters, meeting abstracts, reviews, and editorial articles.

### 2.2 Data extraction

Two reviewers extracted data from each study. The dispute was stable through discussion. Screen the headlines and abstracts of all potentially relevant manuscripts to determine relevance. If the headline and abstract are ambiguous, check the whole article carefully. We collected the information as follows from each study: first author's surname; publication year; type of periodontitis; source of control; geographic region (s); sample capacity; and quantity of subjects with VDR ApaI genotype.

### 2.3 Statistical analysis

The statistics of correlation were evaluated by combining odds ratio (ORs) and 95% confidence interval (CIs). *Z* test was applied to determine the significance of combined ORs and 95% CIs. The heterogeneity was measured by *Q* statistics. In cases of heterogeneity, the random‐effect model was selected to aggregate 95% CIs‐distributed ORs, and then, the fixed‐effect model was applied.

Sensitivity analysis was evaluated by contrasting the results of the fixed‐ and random‐effect models. In addition to comparing all subjects, stratified analysis was conducted according to geographic region(s), control source and type of periodontitis. Statistical analyses were handled by the University City of Texas Stata Corporation (version 10.0; Stata Corporation). *p* < .05 was considered as the statistical significance.

## 3 RESULTS

### 3.1 Description of included studies

**Figure 1:**
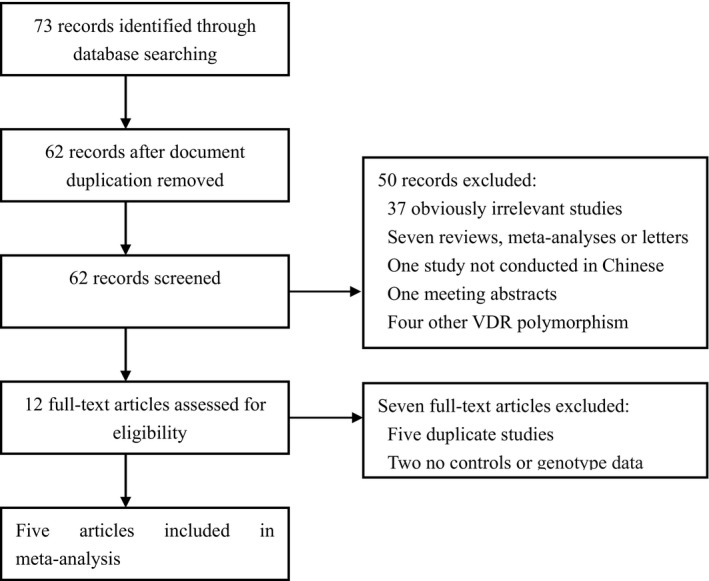
Flow diagram of the literature search

Totally 62 studies that examined the relationship between the VDR polymorphisms and the risk of periodontitis were distinguished after document duplication was deleted in different databases (Figure 1). Fifty articles were excluded after the first screening of titles and abstracts. Of the 12 potential related articles identified for comprehensive research, seven were excluded because of duplication, and no control group or genotype data were available. Finally, five articles (including six case–control studies) (Li et al., 2008; Ma, Zhang, Huang, & Han, 2011; Shao, 2013; Wang et al., 2009; Zhang et al., 2005) measured up the inclusion criteria. The relevant research was published from 2005 to 2013. The meta‐analysis included 734 patients with periodontitis and 687 controls. The control sources of the five studies were population‐based. Characteristics of the studies included are summarized (Table 1).

**Table 1:** Characteristics of studies included in the meta‐analysis

References	Type of periodontitis	Source of controls	Geographic area(s)	Case number	Control number	Cases			Controls			HWE	
						AA	Aa	aa	AA	Aa	aa	*χ* ^2^	*p*
Zhang et al. (2005)	CP	HB	Sichuan	166	80	61	74	31	6	31	43	0.02	.900
Li et al. (2008)	AP	PB	Jiangsu	51	53	15	25	11	16	29	8	0.76	.383
Wang et al. (2009)	CP	PB	Guangdong	107	121	11	51	45	20	46	55	3.51	.061
Ma et al. (2011)	CP	PB	Ningxia	88	92	40	29	19	25	32	35	8.06	.005
Ma et al. (2011)	CP	PB	Ningxia	90	95	25	44	21	28	33	34	8.69	.003
Shao (2013)	CP	PB	Yunnan	232	246	5	11	216	17	4	225	193.08	.000

Abbreviations: HB, hospital‐based; PB, population‐based.

### 3.2 Meta‐analysis

**Figure 2:**
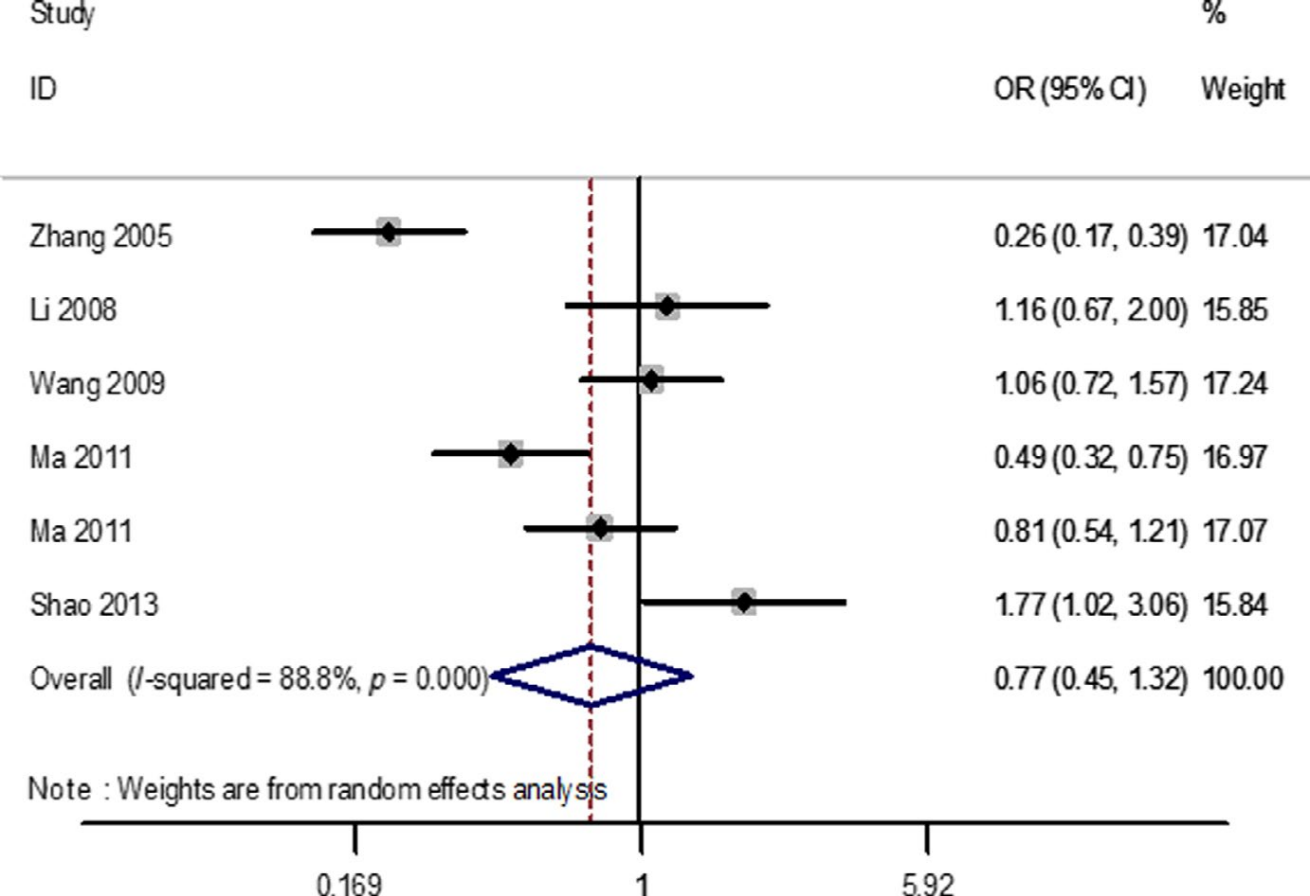
The forest plots of all selected studies on the association between VDR ApaI polymorphism and periodontitis risk in Chinese (for allele model a vs. A)

**Figure 3:**
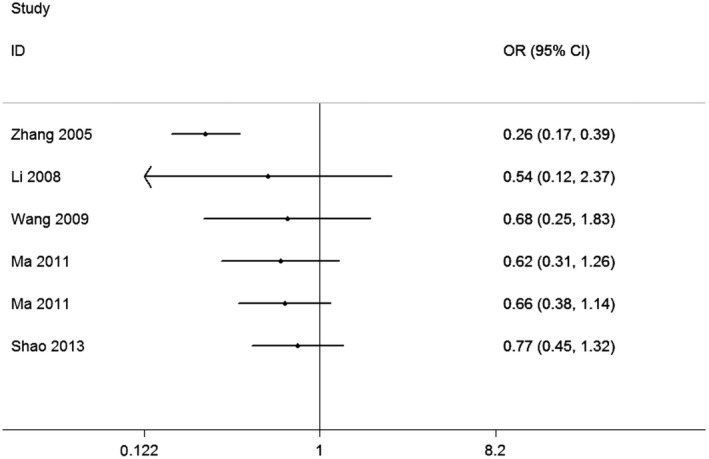
Cumulative analysis of the relationship between VDR ApaI polymorphism and periodontitis risk in Chinese (for allele model a vs. A)

The primary results are enumerated (Table 2). Firstly, heterogeneity analysis was carried out. In the overall analysis, there was no correlation between VDR ApaI polymorphism and periodontitis risk (Figure 2). A cumulative analysis further implied a shortage of correlation between the VDR ApaI polymorphism and the risk of periodontitis among the Chinese population (Figure 3). Subgroup analysis by geographic area (s), control source and periodontitis type, significant correlations between the VDR ApaI periodontitis and variants were found in north China (a vs. A, OR = 0.64, CI = 0.47–0.85; aa vs. AA, OR = 0.48, CI = 0.28–0.82; aa vs. AA + Aa, OR = 0.50, CI = 0.31–0.79).

**Table 2:** Association of the VDR ApaI gene polymorphism on periodontitis susceptibility

Analysis model	*n*	ORr(95%CI)	ORf(95%CI)	*p*_h_
a versus A				
Total analysis	6	0.77 (0.45–1.32)	0.71 (0.59–0.84)	.000
Population‐based	5	0.95 (0.64–1.42)	0.91 (0.75–1.11)	.004
South China	4	0.85 (0.36–2.02)	0.75 (0.60–0.93)	.000
North China	2	0.63 (0.39–1.02)	0.64 (0.47–0.85)	.102
CP	5	0.71 (0.38–1.32)	0.67 (0.55–0.80)	.000
aa versus AA				
Total analysis	6	0.69 (0.25–1.91)	0.61 (0.44–0.84)	.000
Population‐based	5	1.05 (0.49–2.28)	0.95 (0.66–1.38)	.004
South China	4	0.84 (0.16–4.53)	0.70 (0.47–1.05)	.000
North China	2	0.48 (0.24–0.97)	0.48 (0.28–0.82)	.193
CP	5	0.60 (0.19–1.91)	0.56 (0.40–0.79)	.000
aa versus AA + Aa				
Total analysis	6	0.63 (0.35–1.15)	0.60 (0.46–0.77)	.000
Population‐based	5	0.80 (0.52–1.21)	0.78 (0.58–1.04)	.101
South China	4	0.74 (0.29–1.87)	0.65 (0.48–0.89)	.000
North China	2	0.50 (0.31–0.79)	0.50 (0.31–0.79)	.657
CP	5	0.55 (0.30–1.04)	0.56 (0.43–0.73)	.000
aa + Aa versus AA				
Total analysis	6	0.85 (0.39–1.87)	0.75 (0.56–1.00)	.000
Population‐based	5	1.17 (0.62–2.20)	1.05 (0.77–1.45)	.008
South China	4	0.95 (0.26–3.52)	0.79 (0.55–1.15)	.000
North China	2	0.70 (0.29–1.66)	0.69 (0.44–1.07)	.051
CP	5	0.82 (0.32–2.11)	0.72 (0.53–0.97)	.000

Note

North China included Ningxia; South China included Sichuan, Jiangsu, Guangdong, Yunnan.

Abbreviations: Orr, Odds ratio for random‐effect model; ORf, odds ratio for fixed‐effect model; *p*_h_, *p* value for heterogeneity test.

### 3.3 Sensitivity analysis

Statistics analysis was conducted by comparing the results of fixed‐effect and random‐effect models. Except for the allele control model, all significant corresponding ORS remained unchanged, indicating that the outcomes of the meta‐analysis were relatively steady (Table 2).

## 4 DISCUSSION

As an inflammatory disease, periodontitis is most often caused by microorganisms. As a multifactorial disease, previous studies have shown that the susceptibility of individuals to periodontal disease depends partly on genetic factors. The connection between the VDR ApaI polymorphism and periodontitis cause has been attached the attention by many researchers. One meta‐analysis showed that CP patients have a markedly higher frequency of the AA genotype of ApaI (OR, 52.20; 95% CI, 51.39–53.48; *p* < .001) (Deng et al., 2011); however, another meta‐analysis suggested that the ApaI polymorphism has no significant association with susceptibility to periodontitis (CP/AP) (Chen, Li, Zhang, & Wang, 2012). As a result, we proceeded this analysis to more accurately estimate the relationship between VDR‐ApaI polymorphism and periodontitis susceptibility among Chinese population. The analysis involved five case–control studies, including 734 cases of periodontitis and 687 controls. The outcomes showed that VDR‐ApaI polymorphism may be related to the significant reduction of periodontitis risk in North China due to the small sample size.

Lots of studies are conducted to explore the correlation between VDR ApaI variants and periodontitis previously. Several studies (El Jilani et al., 2015; Karasneh et al., 2013; Naito et al., 2007) show that the VDR ApaI polymorphism was related to an increased risk of developing CP, while the opposite results are found with severe generalized CP in Turkish patients (Gunes et al., 2008). The evidence is accordant with our findings, suggesting that the correlation between VDR ApaI variants and periodontitis may be contributed not only to ethnic background, region and sample size, but also to different mechanisms of AP and CP.

This research had clear strengths, such as investigating the influence of geographic area(s) on periodontitis and VDR ApaI risk; however, several shortcomings should be taken notes. First of all, ethnic‐specific meta‐analysis only includes figures on a single ethnic group, so our study applies only to that ethnic group. Secondly, because this meta‐analysis is mainly based on unadjusted impact estimates and cis‐index, the interferences are uncontrolled. Thirdly, the heterogeneity is high and stratified analysis cannot explain it. Other clinical heterogeneities may help to diagnose and classify periodontal diseases, differences in periodontal examinations, and different clinicians; however, due to the limited data available, we cannot investigate these factors. Finally, owing to the limitations of funnel charts, which require a large series of studies, we could not assess publication bias in this meta‐analysis.

All in all, the meta‐analysis suggests that VDR ApaI polymorphism may be connected with a reduced cause of periodontitis in northern China; however, much more researches should be investigated in the future to verify our findings.

## CONFLICT OF INTEREST

The authors declare that they do not have any conflict of interest.

## ETHICAL APPROVAL

This study does not involve any human or animal testing, and all analyses are based on previously published studies; thus, no ethical review and patient consent are required.
